# Overexpression of 7-hydroxymethyl Chlorophyll *a* Reductase from Cucumber in Tobacco Accelerates Dark-Induced Chlorophyll Degradation

**DOI:** 10.3390/plants10091820

**Published:** 2021-08-31

**Authors:** Weikang Liu, Guangling Chen, Jiaqi Chen, Mohammad Shah Jahan, Shirong Guo, Yu Wang, Jin Sun

**Affiliations:** College of Horticulture, Nanjing Agricultural University, Nanjing 210095, China; 2018104123@njau.edu.cn (W.L.); 2019104117@njau.edu.cn (G.C.); 2019104115@njau.edu.cn (J.C.); shahjahansau@gmail.com (M.S.J.); srguo@njau.edu.cn (S.G.)

**Keywords:** cucumber, chlorophyll degradation, darkness, 7-hydroxymethyl chlorophyll *a* reductase (HCAR), leaf senescence

## Abstract

7-hydroxymethyl chlorophyll (Chl) *a* reductase (HCAR) plays critical roles in the Chl cycle and degradation during leaf senescence, however, its function in horticultural crops remains unknown. Here, we identified an *HCAR* gene (*CsHCAR*) from cucumber (*Cucumis sativus* L.) and investigated its roles in response to dark-induced Chl degradation. *CsHCAR* encoded 459 amino acids, which were orthologous to Arabidopsis HCAR, had the conserved domains, and localized in the chloroplast. Gene expression analysis showed that *CsHCAR* expression was the highest in senescent leaves and was responsive to different stresses and phytohormone treatments. Overexpression of *CsHCAR* in tobacco accelerated dark-induced Chl degradation through enhancing the expression of Chl catabolic genes. After 10 d of darkness treatment, the biomass of *CsHCAR* overexpression plants was reduced. Furthermore, the value of net photosynthetic rate, maximum quantum yield of photosystem II, and effective quantum yield of photosystem II in *CsHCAR* overexpression plants was significantly reduced in comparison to that in wild-type (WT) plants. The photosynthetic protein content, including Lhcb1, Lhcb2, Lhcb4, RbcS, and RbcL in *CsHCAR* overexpression plants exhibited a lower level as compared to that observed in WT plants. In addition, the expression of genes encoding these proteins in *CsHCAR* overexpression plants was significantly lower than that in WT plants. Moreover, *CsHCAR* overexpression plants inhibited the dark-induced accumulation of reactive oxygen species (ROS). These results indicate that *CsHCAR* affects the stability of photosynthetic proteins in chloroplasts, positively regulates Chl degradation, and plays an important role in maintaining ROS homeostasis in leaves.

## 1. Introduction

Chlorophyll (Chl) plays a central role in the photosynthetic system and is actively synthesized by glutamate during plant development and degraded into non-fluorescent Chl catabolites during senescence [1]. Chl intermediate molecules are also considered to be important signals of cellular processes, such as the cell cycle [2]. Excessive accumulation of Chl intermediate molecules may result in the production of large amounts of reactive oxygen species (ROS), leading to growth retardation and cell death [3]. As a result, Chl metabolism is strictly regulated by a variety of mechanisms, including gene expression, feedback inhibition, and protein stability [4].

The Chl cycle refers to the mutual transformation between Chl *a* and Chl *b*, which plays vital roles in the aging process [5]. In higher plants, Chl *a* is the degradable form of Chls [6]. The first step of Chl *a* degradation is catalyzed by magnesium (Mg)-dechelatase, encoded by Mendel’s green cotyledon gene, STAY-GREEN (*SGR*), which catalyzes the conversion of Chl *a* to pheophytin *a* (Phetin *a*) [7]. The phytol chain of Phetin *a* is subsequently removed by pheophytinase (PPH) and pheophorbide *a* (Pheide *a*) is produced [8]. The tetrapyrrole ring of Pheide *a* is opened by pheophorbide *a* oxygenase (PAO) to produce red Chl catabolite (RCC) [9]. RCC is subsequently reduced by RCC reductase (RCCR) [10]. The Chl cycle is required to finely regulate the Chl *a*/*b* ratio, which is important for acclimation of plants to the light environment. Chl *b* is synthesized from Chl *a* by the catalyzing action of chlorophyllide *a* oxygenase [11]. When plants need to reduce Chl *b* levels, it is first converted to 7-hydroxymethyl Chl *a* (7-HMChl *a*) by Chl *b* reductase (CBR), which is encoded by non-yellow coloring1 (*NYC1*) and NYC1-like (*NOL*) [12,13]. Then, 7-HMChl *a* is transferred to Chl *a* by 7-hydroxymethyl Chl *a* reductase (HCAR) [14]. However, the Chl cycle is more than a reciprocal conversion of Chl *a* and Chl *b*; it also plays an important role in the degradation of light-harvesting Chl *a*/*b* protein complex of photosystem II (LHCII) [15,16]. Arabidopsis SGR1 physically interacts with the Chl catabolic enzymes (CCEs) and LHCII, forming a multi-protein complex that is likely important for rapid detoxification of Chl catabolic intermediates in senescing chloroplasts [17,18]. Knockout of *NYC1* not only blocks Chl degradation, but also impairs the breakdown of LHCII and the thylakoid membrane during senescence [12,13]. Interestingly, *RCCR*-overexpression plants show increased tolerance to oxidative stress-induced cell death [19], indicating that some CCEs have the potential for controlling cell death mechanisms, possibly through the metabolic channeling of phototoxic Chl intermediates. Arabidopsis HCAR is an orthologous of cyanobacterial divinyl Chl vinyl reductase, which participates in Chl biosynthesis [14,20]. Arabidopsis and rice (*Oryza sativa*) *HCAR* gene mutants show a stay-green phenotype during dark-induced leaf senescence, and the accumulation of Chl decomposition intermediates 7-HMChl *a* and Pheide *a* [14,21]. In addition, HCAR physically interacts with LHCII and other CCEs, such as SGR1, NYC1, NOL, and RCCR, indicating that HCAR is a component of the Chl degradation complex [18]. AtHCAR acts as a limiting factor during Chl cycle and Chl *b* degradation in Chl-*b*-overproducing plants [22]. So far, the research on HCAR is mainly focused on Arabidopsis and rice, however, it has not been reported in horticultural plants.

To identify the *HCAR* gene, we used cucumber as a test material and found that cucumber’s HCAR (*CsHCAR*) amino acid sequence is orthologous to the Arabidopsis; however, its physiological role remains unknown regarding dark-induced Chl degradation. In this study, we performed the functional analysis of *CsHCAR* and found that *CsHCAR* positively regulated dark-induced Chl degradation through increasing Chl catabolic genes (CCGs) expression.

## 2. Results

### 2.1. Identification of Cucumber HCAR

For identification of the *HCAR* gene in cucumber, we used the amino acid sequence of Arabidopsis HCAR (AtHCAR) (AT1G04620) as the probe to perform BLAST research in the cucurbitaceae genome database. Only one putative HCAR sequence was identified, and was named as *CsHCAR*. Further analysis revealed that the *CsHCAR* gene was located on chromosome 3, and the length of coding DNA sequence (CDS) was 1380 bp, encoding 459 amino acids (Table 1). The physicochemical analysis showed that the molecular weight (MW) and theoretical isoelectric point (pI) of *CsHCAR* was 51.19 KDa and 7.54, respectively (Table 1).

**Figure 1:**
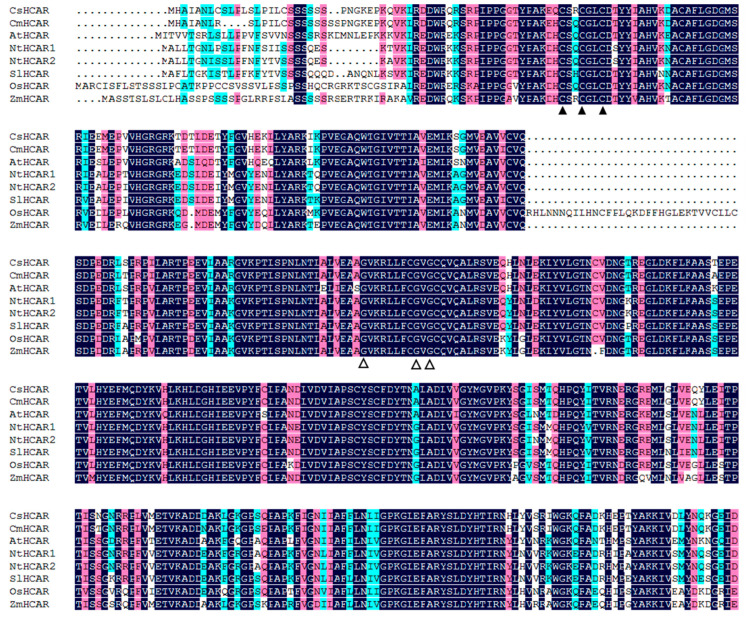
Alignment of the amino acid sequences of HCAR proteins among seven species. Conserved residues are shaded with black. Filled triangles represent the cysteine residues that are predicted to coordinate the iron-sulfur cluster. Open triangles represent the conserved sequence motif in FAD-containing proteins (K×××××G×G). Cs, *Cucumis sativus*; Cm, *Cucumis melo*; At, *Arabidopsis thaliana*; Nt, *Nicotiana tabacum*; Sl, *Solanum lycopersicum*; Os, *Oryza sativa*; Zm, *Zea mays*.

In order to further investigate the evolutionary relationship between *CsHCAR* and other species, we searched the HCAR proteins of 35 species in the NCBI database (Table S1). After multiple sequence alignment of the 35 HCAR protein sequences using the Clustal W method in MEGA 5.0 software, a phylogenetic tree was constructed with the Neighbor-joining method. The phylogenetic tree analysis showed that the HCAR proteins were divided into four groups, and the *CsHCAR* was orthology with those in cucurbitaceous plants (Figure S1). Most of the plants had only one HCAR protein, however, a few plants had evolved two HCAR members (Figure S1 and Table S1). Amino acid sequence alignment showed that the HCAR protein structure of cucumber, melon (*Cucumis melo*), Arabidopsis, tobacco (*Nicotiana tabacum*), rice, tomato (*Solanum lycopersicum*), and maize (*Zea mays*) was highly conserved and had the same domains, such as the cysteine residues and motif in FAD-containing proteins (Figure 1), indicating that they might have the same functions.

### 2.2. Analysis of CsHCAR Expression Profiles and Subcellular Localization

**Figure 2:**
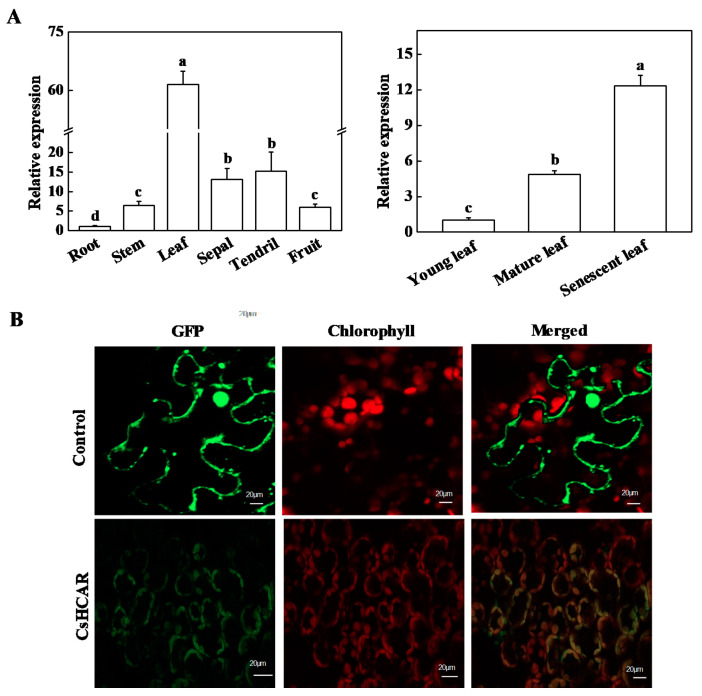
Expression patterns of *CsHCAR* in different tissues and its subcellular localization. (**A**) qPCR analysis of the expression of *CsHCAR* in roots, stems, leaves, fruits, sepals, and tendrils of cucumber. The expression level in roots or young leaf was set to 1.0. The results represent the mean ± SE (*n* = 3). Means with the same letter did not significantly differ at *p* < 0.05 according to Tukey’s test. (**B**) Subcellular localization of *CsHCAR* in tobacco cells. Bars: 20 μm.

In order to test the expression of *CsHCAR* in different tissues, cucumber roots, stems, young leaves, mature leaves, senescent leaves, fruits, sepals, and tendrils were used as materials for quantitative real-time PCR (qPCR) analysis. As shown in Figure 2A, *CsHCAR* expression was mainly concentrated in leaves, and also expressed in sepals, stems, fruits, and tendrils. The expression levels of *CsHCAR* in mature leaves and senescent leaves were 3.8-fold and 11.4-fold higher than those in young leaves, respectively (Figure 2A), and higher expression levels were observed in senescent leaves, indicating that *CsHCAR* might play an important regulatory role in the senescence process of cucumber plants.

To determine the localization of *CsHCAR* in cells, *Agrobacterium tumefaciens* harboring the pFGC5941-**CsHCAR*-GFP* fusion expression vector was infiltrated into *Nicotiana benthamiana* leaves, and the fluorescence of GFP was observed by laser confocal microscope. As shown in Figure 2B, the fluorescence of *CsHCAR*-GFP fusion protein was overlapped with the chloroplast auto-fluorescence signal, indicating that the *CsHCAR* protein was localized in the chloroplast.

### 2.3. Response of *CsHCAR* to Multiple Phytohormones and Abiotic Stresses

**Figure 3:**
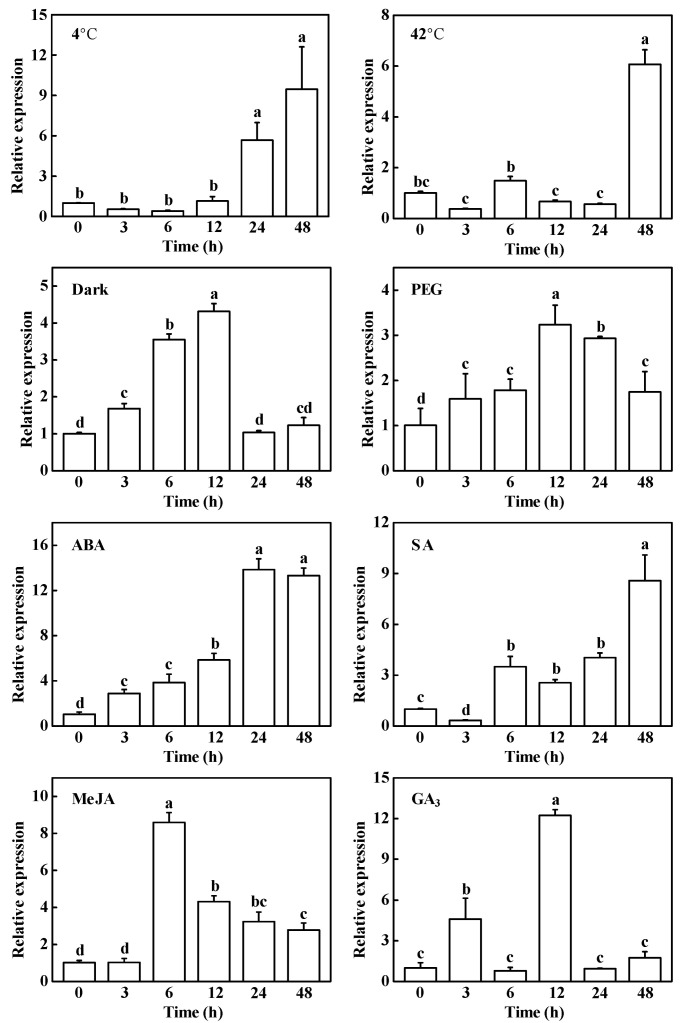
Expression patterns of *CsHCAR* in cucumber leaves under different abiotic stresses and phytohormone treatments. 4 °C, 42 °C, dark, and PEG represented cucumber plants treated with cold, heat, darkness, and drought stress, respectively. ABA, SA, MeJA, and GA_3_ represented cucumber foliar treated with 100 μM ABA, 100 μM SA, 100 μM MeJA, and 100 μM GA_3_, respectively. The leaf samples were collected at the indicated time points and analyzed by qPCR. The results represent the mean ± SE (*n* = 3). Means with the same letter did not significantly differ at *p* < 0.05 according to Tukey’s test.

It has been demonstrated that environmental stresses and phytophormones mediate Chl degradation in plants [3,6]. Considering the critical role of HCAR in Chl breakdown, we analyzed the response of *CsHCAR* to cold, heat, dark, drought stress, abscisic acid (ABA), salicylic acid (SA), methyl jasmonate (MeJA), and gibberellin (GA_3_), respectively. Cold, heat stress, and SA treatment had similar effects on *CsHCAR*, and the expression level of *CsHCAR* was firstly decreased and then increased, and reached the highest level at 48 h, which increased by approximately 8.5-fold, 5.1-fold, and 7.6-fold, respectively (Figure 3). Under drought and darkness treatment, the expression of *CsHCAR* increased rapidly in a short time and reached the highest level at 12 h (Figure 3). In the case of exogenous GA_3_ treatment, *CsHCAR* expression was rapidly up-regulated within 3 h, recovered to the pre-treatment level at 6 h, and reached the highest level at 12 h, increasing by about 11.2 times compared with 0 h (Figure 3). Under ABA treatment, the expression of *CsHCAR* increased continuously and reached the highest level at 24 h, which increased by about 12.9 times (Figure 3). Under exogenous MeJA treatment, the expression of *CsHCAR* was firstly increased and then decreased, and reached the highest level at 6 h, which was about 7.6 times higher than that of the control plants (Figure 3). Therefore, *CsHCAR* could be induced by different stresses and phytohormone treatments, suggesting that it might play a critical role in environmental stresses- and phytohormones-induced Chl degradation.

### 2.4. Overexpression of CsHCAR Promotes Chl Degradation

In order to investigate the function of *CsHCAR*, the *Agrobacteria*-mediated leaf plate method was used to perform genetic transformation in tobacco to obtain the transgenic plants that were overexpressing the *CsHCAR*. The rooting plants were screened by hygromycin, the DNA was extracted and verified by PCR, and two independent overexpression positive lines were identified (Figure S2A). Immunoblotting analysis revealed that these two lines expressed high *CsHCAR* protein levels (Figure S2B). Therefore, OE-1# and OE-2# lines were selected for subsequent experiments.

**Figure 4:**
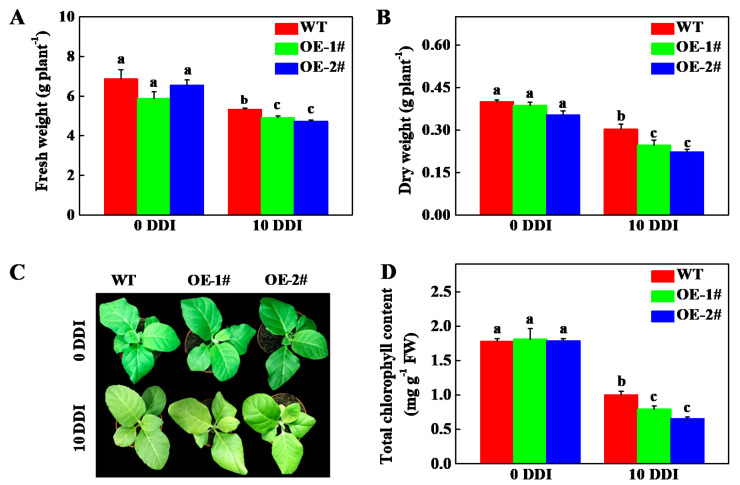
Analysis of dark-induced chlorophyll degradation of transgenic tobacco overexpression of *CsHCAR*. (**A**) Fresh weight; (**B**) dry weight; (**C**) tobacco seedlings phenotype; (**D**) Total chlorophyll content. The results represent the mean ± SE (*n* = 3). Means with the same letter did not significantly differ at *p* < 0.05 according to Tukey’s test. WT, wild-type; OE-1# and OE-2#, 2 independent transgenic tobacco overexpression of *CsHCAR*; DDI, day(s) of dark incubation; FW, fresh weight.

**Figure 5:**
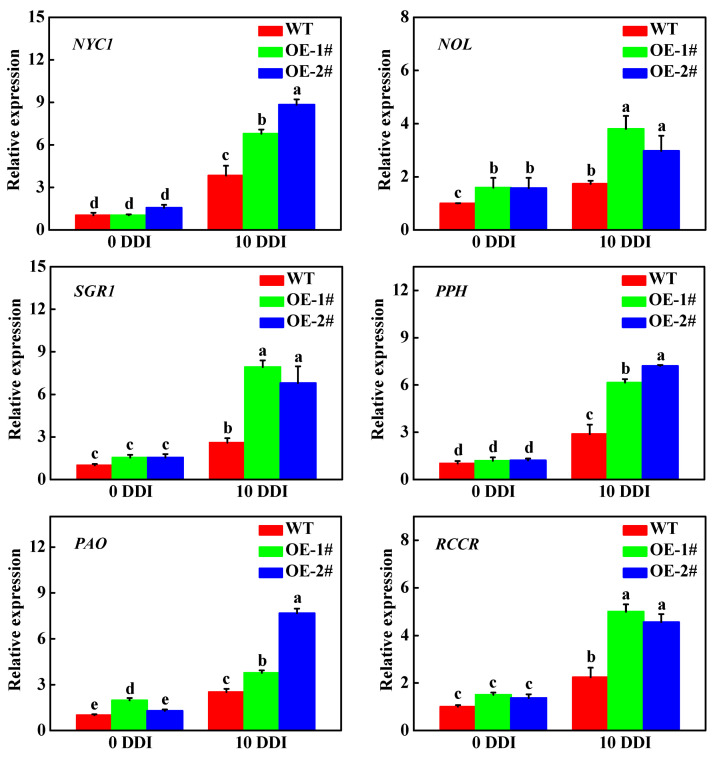
Expression of chlorophyll degradation related genes in leaves of wild-type and *CsHCAR* overexpression tobacco seedlings. The results represent the mean ± SE (*n* = 3). Means with the same letter did not significantly differ at *p* < 0.05 according to Tukey’s test. WT, wild-type; OE-1# and OE-2#, 2 independent transgenic tobacco overexpression of *CsHCAR*. DDI, day(s) of dark incubation; *NYC1*, non-yellow coloring1; *NOL*, NYC1-like; *SGR1*, stay-green1; *PPH*, pheophytinase; *PAO*, pheophorbide *a* oxygenase; *RCCR*, red chlorophyll catabolite reductase.

After darkness treatment for 10 d, the fresh weight (FW) and dry weight (DW) of *CsHCAR* overexpression plants were significantly lower than that of the wild-type (WT) plants (Figure 4A,B). After 10 d of darkness treatment, all of the plants showed different degrees of yellowing; however, the overexpression lines had wrinkle and more obvious chlorosis of the leaves (Figure 4C). The total Chl content in WT plants decreased by 44.06%, while OE-1# and OE-2# lines decreased by 56.11% and 63.48%, respectively, in comparison to the control plants (Figure 4D). To further verify the function of *CsHCAR* in Chl degradation, we detected the expression level of CCGs in WT and *CsHCAR* overexpression plants. Although darkness induced the expression of CCGs in WT and *CsHCAR* overexpression plants, their expression levels in *CsHCAR* overexpression plants were significantly higher than that in WT plants (Figure 5). Therefore, overexpression of *CsHCAR* promoted dark-induced Chl degradation through upregulating the expression of CCGs.

### 2.5. Overexpression of CsHCAR Affects Photosynthesis

**Figure 6:**
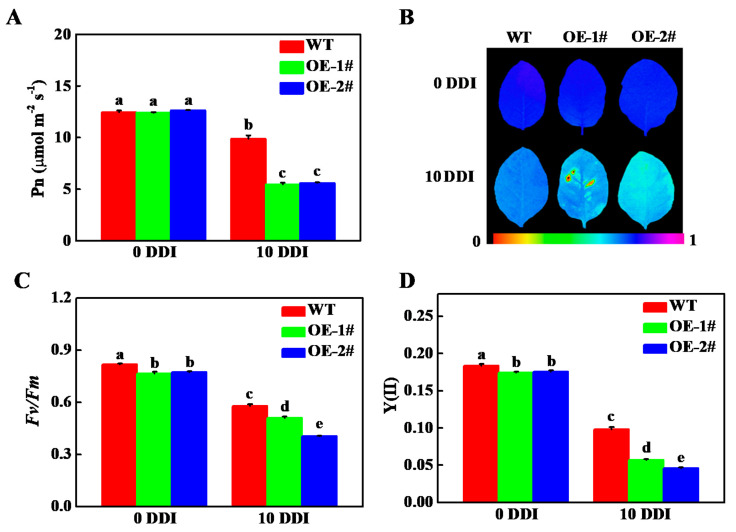
Effects of darkness on the photosynthesis and chlorophyll fluorescence of *CsHCAR* overexpression tobacco seedlings. (**A**) Net photosynthetic rate (Pn). (**B**,**C**) The maximum quantum yield of photosystem II (*Fv/Fm*). (**D**) The effective quantum yield of photosystem II [Y(II)]. The results represent the mean ± SE (*n* = 3). Means with the same letter did not significantly differ at *p* < 0.05 according to Tukey’s test. WT, wild-type; OE-1# and OE-2#, 2 independent transgenic tobacco overexpression of *CsHCAR*; DDI, day(s) of dark incubation.

In order to test the effect of *CsHCAR* on photosynthesis, we compared the net photosynthetic rate (Pn) of *CsHCAR* overexpression and WT plants. No significant difference was observed between WT and *CsHCAR* overexpression plants under normal growth conditions, while WT plants showed higher Pn under darkness stress (Figure 6A). Darkness induced the decrease in the maximum quantum yield of photosystem II (*Fv/Fm*) and effective quantum yield of photosystem II [Y(II)] in all of the plants; however, the values of *Fv/Fm* and Y(II) in WT plants were significantly higher than those in *CsHCAR* overexpression plants at 10 d of darkness treatment (Figure 6B–D). The dark-induced photosystem II damage was more serious in *CsHCAR* overexpression tobacco plants as a result of reduction in photosynthesis efficiency.

**Figure 7:**
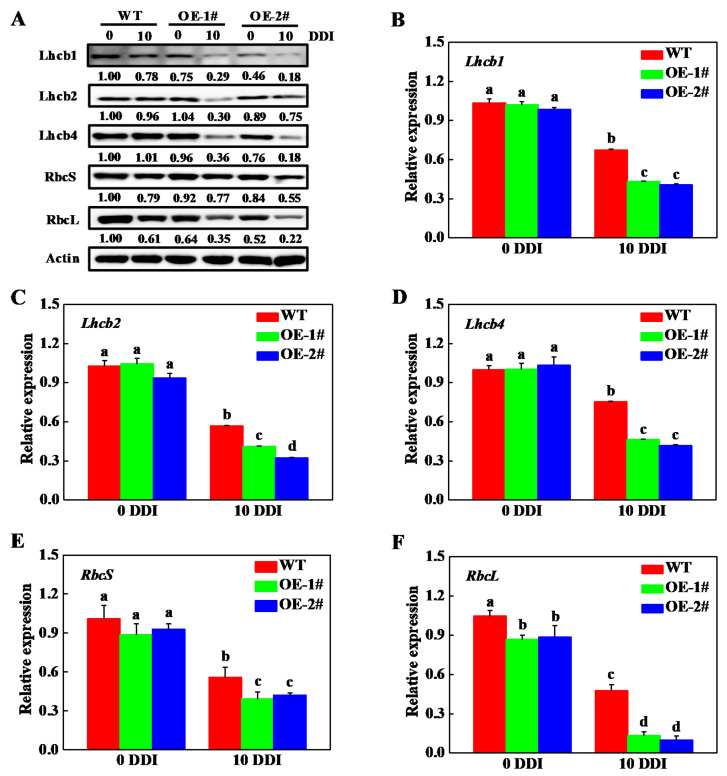
The abundance of photosystem proteins and the expression of related genes in *CsHCAR* overexpression transgenic tobacco seedlings. (**A**) Immunoblotting analysis of the protein abundance of photosystem proteins. The band intensity was quantified using Image J software to calculate to the protein level relative to the 0 d of WT plants, which was set to 1. The relative protein level is shown under the band; (**B**) the expression of *Lhcb1*; (**C**) the expression of *Lhcb2*; (**D**) the expression of *Lhcb4*; (**E**) the expression of *RbcS*; (**F**) the expression of *RbcL*. The results represent the mean ± SE (*n* = 3). Means with the same letter did not significantly differ at *p* < 0.05 according to Tukey’s test. WT, wild-type; OE-1# and OE-2#, 2 independent transgenic tobacco overexpression of *CsHCAR*; DDI, day(s) of dark incubation.

To further investigate the role of *CsHCAR* on photosynthesis, the differences in the content of photosystem proteins were analyzed by immunoblotting. As shown in Figure 7A, the protein levels of Lhcb1, Lhcb2, Lhcb4, RbcS, and RbcL in WT plants were higher than those in *CsHCAR* overexpression plants under normal conditions. Although the protein abundances of Lhcb1, Lhcb2, RbcS, and RbcL in WT and *CsHCAR* overexpression plants were decreased under darkness treatment, the levels of these proteins in WT plants were higher than those in *CsHCAR* overexpression plants (Figure 7A). The abundances of Lhcb4 in *CsHCAR* overexpression plants were also lower than those observed in WT plants after 10 d of darkness stress (Figure 7A). Furthermore, the expression of genes encoding these proteins in the plants was restrained, however, their expression levels in WT plants were still higher than those in *CsHCAR* overexpression plants (Figure 7B–F). These results indicated that the decline of photosynthesis efficiency in *CsHCAR* overexpression plants might result from the lower abundance of photosystem proteins and decreasing the expression of genes encoding these proteins.

### 2.6. Overexpression of CsHCAR Reduces ROS Production

**Figure 8:**
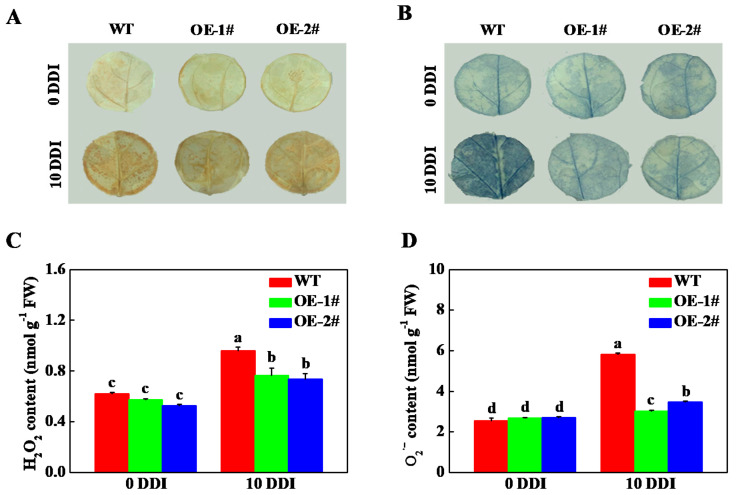
Overexpression of *CsHCAR* inhibits the accumulation of reactive oxygen species. (**A**) DAB staining; (**B**) NBT staining; (**C**) H_2_O_2_ content in leaves; (**D**) O_2_^•−^ content in leaves. The results represent the mean ± SE (*n* = 3). Means with the same letter did not significantly differ at *p* < 0.05 according to Tukey’s test. WT, wild-type; OE-1# and OE-2#, 2 independent transgenic tobacco overexpression of *CsHCAR*; DDI, day(s) of dark incubation; FW, fresh weight.

Cell death is often accompanied by an increase in ROS level [23]. Previous studies on HCAR are mainly focused on plant senescence, and there are few reports on the effect of HCAR on the content of ROS in leaves. Therefore, we compared the accumulation and production of H_2_O_2_ and O_2_^•−^ in leaves of WT and *CsHCAR* overexpression tobacco seedlings. 3,3′-diaminobenzidine (DAB) and nitroblue tetrazolium (NBT) histochemical staining results showed that H_2_O_2_ and O_2_^•−^ were accumulated in the leaves of all plants after 10 d of darkness stress, while ROS accumulation in leaves of WT plants was higher than that of *CsHCAR* overexpression seedlings (Figure 8A,B). Furthermore, the content of H_2_O_2_ and O_2_^•−^ in *CsHCAR* overexpression was significantly lower than that in WT plants, which was consistent with the results of tissue staining (Figure 8C,D). These results suggested that overexpression of *CsHCAR* inhibited the accumulation of ROS, which might alleviate dark-induced cell death.

## 3. Discussion

Chl is a potential molecule for the production of ROS, and it is converted into non-fluorescent Chl decomposition metabolites during aging [3,24]. Disorder of Chl metabolism will lead to the accumulation of intermediate molecules, which will cause necrotic changes in plant leaves [1,25]. However, when Chl supply is limited, photosynthetic activity is reduced, leading to plant growth retardation [26]. Therefore, it is very important to strictly control the synthesis and degradation of Chl during greening and senescence. The Chl cycle plays an important role in maintaining the balance of Chl. This reciprocal transformation pathway between Chl *a* and Chl *b* is called the Chl cycle, which is controlled by CBR and HCAR [2,12,14]. At present, studies on HCAR are mainly focused on Arabidopsis and rice, and it has not been reported in horticultural plants. In this study, we found that there was one *HCAR* gene in the cucumber genome, which was located on chromosome 3 and encoded 459 amino acids (Table 1). Amino acid sequence comparison showed that the protein structure of HCAR in cucumber was highly conserved and had the same domain as other species (Figure 1). The amino acid sequence of cucumber HCAR was orthologous to that of Arabidopsis, suggesting that it may have a similar function to Arabidopsis. In Arabidopsis, HCAR catalyzes the conversion of 7-HMChl *a* to Chl *a*, and *HCAR* gene mutants show delaying Chl degradation, while its overexpression plants accelerate leaf yellowing in dark-induced senescence [14,18]. Furthermore, rice *hcar* mutant also displays persistent green phenotype in dark-induced and natural senescence [21]. Here, we found that *CsHCAR* was induced by darkness, and highly expressed in senescent leaves (Figure 2A and Figure 3). Furthermore, leaves of tobacco plants that were overexpressing *CsHCAR* turned yellow, the Chl degradation rate was accelerated, and the CCGs were significantly up-regulated during darkness treatment (Figure 4 and Figure 5), indicating that up-regulating *CsHCAR* promotes Chl degradation and its function is similar to *AtHCAR* and *OsHCAR*.

Phytohormones play critical roles in regulating leaf senescence [24]. Environmental stresses accelerate leaf senescence, accompanied by the production of ABA, SA, and JA [27,28,29,30]. It has been demonstrated that ABA, SA, and JA positively regulate leaf senescence through activating the expression of CCGs [31,32,33]. Transcription factors in the downstream of these phytohormones, such as ABF3, ABF4, ABI5, MYC2, MYC3, and MYC4, directly bind to the promoters of *NYC1*, *PPH*, and *PAO* to trigger their expression, resulting in promoting Chl breakdown [32,34,35]. In this study, we found that *CsHCAR* was induced by foliar application of ABA, SA, and MeJA, suggesting that it might mediate these phytohormones-induced Chl degradation. In addition, HCAR physically interacts with CCEs and LHCII to form a complex, which plays critical roles in Chl degradation [18,21]. Crystal structure analysis reveals that AtHCAR has the potential to form trimers, which may be critical for its interaction with LHCII [36]. HCAR can form the dimer or trimer through interaction with LHCII and other CCEs, which might enhance its functions [18,21]. We found that the protein abundances of Lhcb1, Lhcb2, and Lhcb4 in *CsHCAR* overexpression tobacco plants were reduced and lower than those in WT plants under darkness stress (Figure 7A). However, the stability of the photosystem protein in *oshcar* mutants was higher than that in WT plants [21]. These results indicated that the gene abundance of *HCAR* was proportional to the degradation rate of Chl and photosystem protein in senescent leaves.

It is reported that silencing or knockout of CCG, such as *PAO* and *RCCR*, exhibits an accelerated cell death phenotype via accumulation of excess phototoxic Chl intermediates [9,37]. HCAR may be involved in regulating cell death signaling by regulating the metabolic process of Chl degradation [25]. Cell death is often accompanied by an increase in ROS levels [23]. It was found that after 10 d of darkness stress, ROS accumulation in leaves of WT plants was significantly higher than that of *CsHCAR* overexpression tobacco seedlings (Figure 8). *Athcar* and *oshcar* mutants accumulate more ROS than WT plants, while their overexpression plants maintain lower levels of ROS [21]. Furthermore, knockout of *HCAR* promotes the accumulation of 7-HMChl *a* and Pheide *a* [14,21]. WT and *hcar* mutant protoplasts treated with 7-HMChl *a* or Pheide *a* both induce singlet oxygen production, however, the intensity in *hcar* mutant protoplasts is more obvious [21]. Thus, the accumulation of 7-HMChl *a* and Pheide *a* incudes the production of singlet oxygen to trigger cell death. During natural senescence and dark-induced senescence, overexpression of *HCAR* can alleviate the symptoms of non-apoptotic programmed death in plants with excessive accumulation of Chl *b* [22]. Therefore, these results suggested that *CsHCAR* might play an important role in the regulation of leaf cell death.

## 4. Materials and Methods

### 4.1. Plant Material and Growth Conditions

Cucumber (*Cucumis sativus* L. cv Jinchun No. 2) was used as the test material and the seeds were purchased from Tianjin Kernel Cucumber Research Institute (Tianjin, China) and used in our experiments. The uniformly germinated seeds were sown in plastic pots (10 cm × 7 cm × 8 cm) covered with a mixture of peat and vermiculite (2:1, *v*:*v*). The growth conditions were maintained as follows: 14/10 h light/dark cycle, 25/18 °C day/night, 75–80% relative humidity, and 300 µmol m^−2^ s^−1^ photosynthetic photon flux density (PPFD).

To analyze the possible effect of phytohormones and stress on *CsHCAR* expression, cucumber seedlings at the third leaves stage were treated with different phytohormones and imposed seedlings to the different abiotic stresses. For phytohormones treatment, 100 μM ABA, SA, MeJA, and GA_3_ were sprayed on cucumber leaves, respectively. Cucumber plants were treated with 4 °C as cold stress, 42 °C as heat stress, 20% polyethylene glycol 6000 (PEG) as drought stress, and 48 h darkness as dark stress. The leaf samples were collected at various time points (0, 3, 6, 12, 24, and 48 h).

WT and transgenic tobacco seeds were sown on a plate containing Murashige & Skoog solid medium, and, after germination, seedlings were transferred into seedling substrate (10 cm × 7 cm × 8 cm). When the seedlings grew to 4 leaves, WT and transgenic tobacco seedlings with the same growth character were selected and cultured in darkness for 10 d. Seedlings grown with normal light were used as the control. After 10 d of darkness treatment, the seedlings were sampled and frozen with liquid nitrogen and then stored in the −80 °C.

### 4.2. Identification and Sequence Analysis of HCAR

To identify *CsHCAR*, BLAST, a search of the cucurbitaceae genome database (http://cucurbitgenomics.org/ (accessed on 3 August 2021)) was performed with Arabidopsis HCAR amino acid sequence. The protein MW and pI were analyzed using Protparam (http://web.expasy.org/protparam/ (accessed on 3 August 2021)). The conservative structure of the protein domains was analyzed using Pfam database (http://pfam.xfam.org/ (accessed on 3 August 2021)). The phylogenetic tree of a set of HCAR protein sequences, searched in the NCBI database, was constructed using MEGA 5.0 software with Neighbor-joining method.

### 4.3. Total RNA Extraction and Gene Expression Analysis

Total RNA was extracted using RNA simple Total RNA Kit (Tiangen, Beijing, China) according to the manufacturer’s instruction. The total RNA (1 µg) was reverse transcribed using the HiScript II Q RT SuperMix for qPCR (+gDNA wiper) Kit (Vazyme, Nanjing, China). qPCR assays were performed using the StepOnePlus™ Real-Time PCR System (Applied Biosystems, Foster, CA, USA) with the ChamQ SYBR qPCR Master Mix (Vazyme, Nanjing, China). The PCR program consisted of predenaturation at 95 °C for 5 min, followed by 40 cycles of 95 °C for 10 s, and 60 °C for 30 s. Specific primers (Table S2) were designed according to the CDS. The *actin* gene was used as an internal control. The relative gene expression was calculated based on the mean of three biological replications and were calculated using the 2^−ΔΔCT^ method [38].

### 4.4. Subcellular Localization of HCAR

The full-length CDS of *CsHCAR* was amplified with specific primers (Table S3) and inserted into pFGC5941-GFP vector to generate a *CsHCAR*-GFP fusion expression vector, then transformed into the *N. benthamiana* leaves using *A. tumefaciens* strain GV3101. Control samples were transformed with an empty pFGC5941-GFP vector. After inoculation for 2 d, the GFP fluorescence was monitored under an LSM 800 confocal microscope (Zeiss, Oberkochen, Germany). All transient expression assays were repeated at least three times.

### 4.5. Plasmid Construction and Screening Transgenic Plants

To generate the *CsHCAR* overexpression construct, the full-length CDS was amplified with specific primers (Table S3) using cucumber cDNA as the template. The PCR product was inserted into the plant transformation vector pFGC1008-HA using the ClonExpress II One Step Cloning Kit (Vazyme, Nanjing, China). The constructed plasmid was transformed into *A. tumefaciens* strain EHA105, then transferred into NC89 tobacco plants as described previously [39]. Transgenic plants that were overexpressing the *CsHCAR* were identified by genomic PCR and immunoblotting. Two independent homozygous lines of the T_2_ progeny (OE-1# and OE-2#, overexpression line 1 and 2) were used for further experiments.

### 4.6. Protein Extraction and Immunoblotting Analysis

Total proteins were extracted from tobacco seedlings leaves (0.5 g) as previously described [40]. The samples were ground in liquid nitrogen and homogenized with 600 μL extraction buffer [50 mM Tris-HCl (pH 6.8), 2 mM EDTA, 2%, SDS, 10% glycerol, and 6% β-mercaptoethanol] and cleared by centrifugation (12,000× *g* for 30 min) at 4 °C. The protein concentrations were detected using BCA Protein Assay Kit (Fude Biological Technology CO., LTD., Hangzhou, China). Then, the samples were boiled at 99 °C with 2 × SDS loading buffer for 10 min.

For immunoblotting, the denatured proteins were separated using 12% sodium dodecyl sulfate-polyacrylamide gel electrophoresis (SDS-PAGE), followed by electro-blotting transferred to a polyvinylidene difluoride membrane (Millipore, Bedford, MA, USA). The membrane was blocked for 1 h in TBST buffer (20 mM Tris, 150 mM NaCl, 0.05% Tween-20, pH 7.6) with 5% skim milk powder at room temperature. Then, the membrane was probed with a mouse anti-HA antibody (Thermofisher, Rockford, IL, USA), a rabbit anti-Lhcb1 antibody (Agrisera, Vännäs, Sweden), a rabbit anti-Lhcb2 antibody (Agrisera), a rabbit anti-Lhcb4 antibody (Agrisera), a rabbit anti-RbcS antibody (Agrisera), a rabbit anti-RbcL antibody (Agrisera), or a rabbit anti-actin antibody (Abcam, Cambridge, UK). Subsequently, the membranes were incubated in TBST buffer containing a goat anti-mouse HRP-linked antibody (Abmart, Shanghai, China) or goat anti-rabbit HRP-linked antibody (Cell Signaling Technology, Beverly, MA, USA), and the complexes on the blot were visualized using the FDbio-Femto ELC Kit (Fude Biological Technology CO., LTD., Hangzhou, China). The intensity of bands was quantified using Image J software (National Institutes of Health, Bethesda, MD, USA).

### 4.7. Measurement of Chl Content

The Chl was extracted from the fifth leaves (0.2 g) and the samples were shredded and soaked in 25 mL ethanol in the darkness until completely whitened. Then, Chl concentrations were measured by spectrophotometry at 665 and 649 nm according to the method previously described [41].

### 4.8. Determination of Growth, Chl Fluorescence Parameters and Pn

For measurement of the FW and DW of tobacco seedlings, the plants were washed with distilled water and dried with absorbent paper after darkness stress for 10 d. The FW was measured by electronic balance (Sartorius, Goettingen, Germany). After that, the whole plants were enclosed in the envelopes and placed in an oven (Shanghai Yiheng Scientific Instrument Co., Ltd., Shanghai, China) at 105 °C for 15 min. Afterward, the oven temperature was adjusted to 75 °C to obtain a constant DW.

Tobacco plants were dark-adapted for 30 min to measure the Chl fluorescence parameters with the portable fluorometer (PAM-2100, Heinz Walz, Effeltrich, Germany) according to previously described method [42].

A portable photosynthetic apparatus LI-6400 (LI-COR, Lincoln, NE, USA) was used to measure the Pn of tobacco leaves, as in the method described by Zhang et al. [43]. For the measurement of data, the cuvette conditions were provided as PPFD of 800 μmol m^−2^ s^−1^, relative humidity at 60–70%, temperature of 25 °C, and CO_2_ concentration of 380 ± 10 μmol mol^−1^.

### 4.9. Measurement of ROS Content and Histochemical Staining

The H_2_O_2_ concentration in leaves was estimated by a method described previously [44]. The superoxide anion (O_2_^•−^) production was determined as previously described [45]. Tobacco leaves treated with darkness for 0 d and 10 d were cut into 1.5 cm leaf discs for H_2_O_2_ and O_2_^•−^ staining. DAB staining method was used for H_2_O_2_ histochemical staining [46]. After soaking tobacco leaf plates in 50 mM Tris-HCl (pH 3.8) solution containing 1 mg L^−1^ DAB, they were subjected to light for staining until the brown spots appeared on the leaves. O_2_^•−^ histochemical staining was performed using NBT [46]. After soaking tobacco leaves in 25 mM HEPES (pH 7.8) solution containing 1 mg L^−1^ NBT, they were dyed in the dark for 3–5 h. Decolorization was carried out in boiling water bath with 95% ethanol until Chl was completely removed from the leaves.

### 4.10. Statistical Analysis

Experimental data were analyzed using the SPSS statistics software IBM SPSS statistics 20.0 (SPSS Inc., Chicago, IL, USA). Statistically significant differences among the treatments were determined using Tukey’s test at *p* < 0.05. Three independent biological replicates were used for each determination.

## 5. Conclusions

In conclusion, we identified one *HCAR* gene in the cucumber genome, which was orthologous with other species, including Arabidopsis, melon, and rice. The gene expression levels of *CsHCAR* in mature leaves and senescent leaves were higher, indicating that it might play an important regulatory role in the senescence process of cucumber. Overexpression of *CsHCAR* promoted dark-induced Chl degradation through elevating the transcription of CCGs. The photosynthesis efficiency was also hampered in *CsHCAR* overexpression plants by the reduction of *Fv/Fm* and suppression the light-catching antenna protein content, as well as their encoding gene expression. Furthermore, the ROS accumulation in leaves of *CsHCAR* overexpression lines significantly decreased after 10 d of darkness treatment. These results indicated that *CsHCAR* affected the stability of chloroplast photosynthetic proteins, and positively regulated Chl degradation.

## Figures and Tables

**Table 1:** Properties of the cucumber HCAR.

Name	Accession Number	Location in Chromosome	CDS (bp)	Amino Acid	MW (KDa)	pI
HCAR	CsaV3_3G011480	Chr3	1380	459	51.19	7.54

Cucumber HCAR was identified from the cucurbitaceae genome database (http://cucurbitgenomics.org/ (accessed on 3 August 2021)) through BLAST research using the amino acid sequence of Arabidopsis HCAR (AT1G04620) as the probe. The protein MW and pI were analyzed using Protparam (http://web.expasy.org/protparam/ (accessed on 3 August 2021)). HCAR, 7-hydroxymethyl chlorophyll *a* reductase; Chr, chromosome; CDS, coding DNA sequence; bp, base pair; MW, molecular weight; pI, isoelectric point.

## Data Availability

Not applicable.

## Supplementary Materials

The following are available online at https://www.mdpi.com/article/10.3390/plants10091820/s1, Figure S1: Phylogenetic tree of CsHCAR protein from cucumber and other species. Figure S2: Identification of the *CsHCAR* overexpression transgenic tobacco plants. Table S1: GenBank accession numbers of HCAR used to build phylogenetic tree. Table S2: Primers used for qPCR assays. Table S3: Primer sequences for vector construction.
