# Supplementary material for: Overexpression of 7-hydroxymethyl Chlorophyll a Reductase from Cucumber in Tobacco Accelerates Dark-Induced Chlorophyll Degradation
Source: Plants (Basel). 2021 Aug 31;10(9):1820. doi: 10.3390/plants10091820 (PMC8465020; doi:10.3390/plants10091820)
Supplement: Supplementary file 1 [file plants-10-01820-s001.zip › Supplementary Materials/Supplemental Figures.pdf]

# Figure S1

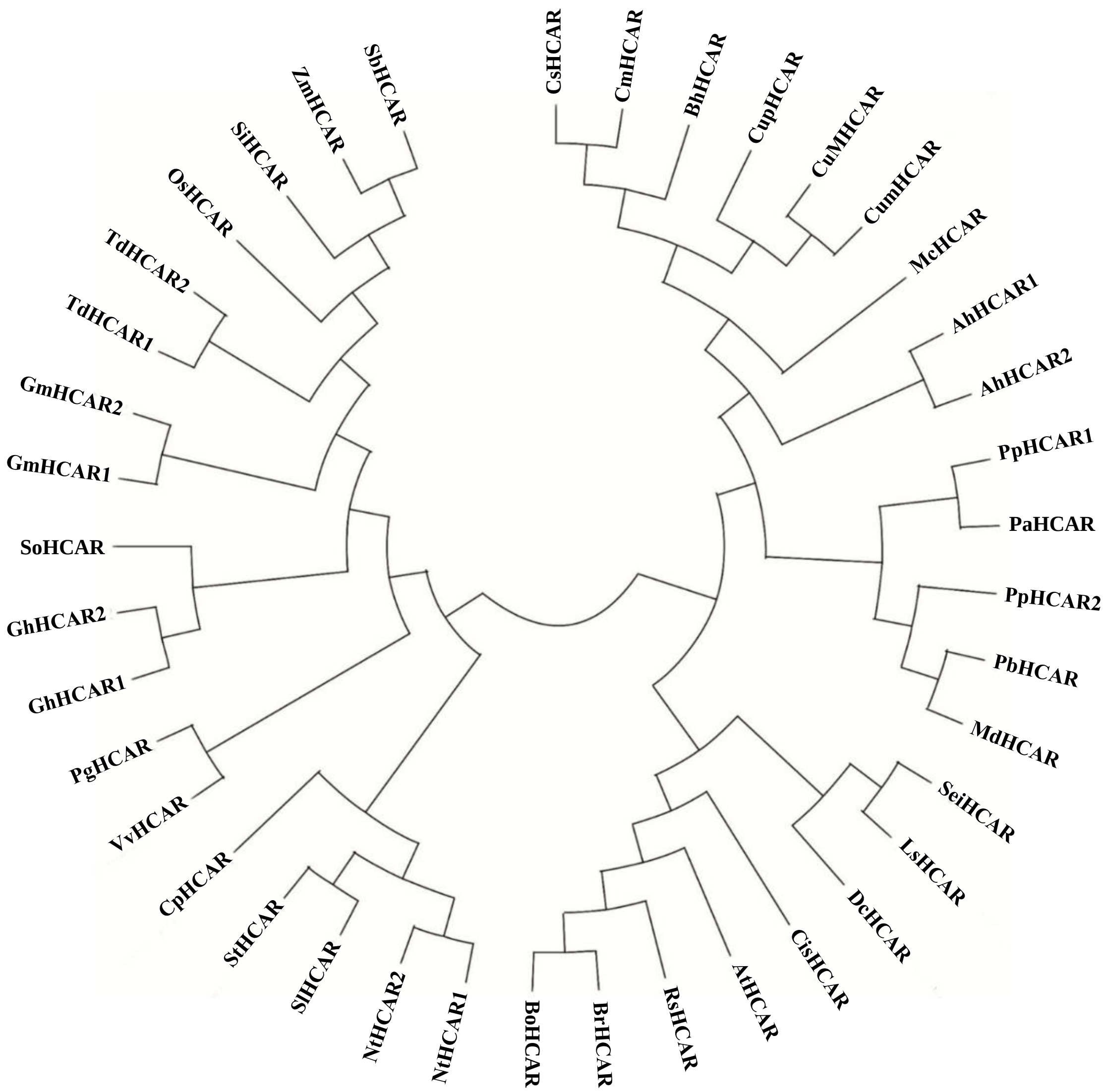


**Figure S1.** Phylogenetic tree of CsHCAR protein from cucumber and other species. Ah, *Arachis hypogaea*; At, *Arabidopsis thaliana*; Bh, *Benincasa hispida*; Bo, *Brassica oleracea*; Br, *Brassica rapa*; Cis, *Citrus sinensis*; Cm, *Cucumis melo*; Cp, *Carica papaya*; Cs, *Cucumis sativus*; CuM, *Cucurbita maxima*; Cum, *Cucurbita moschata*; Cup, *Cucurbita pepo*; Dc, *Daucus carota*; Gh, *Gossypium hirsutum*; Gm, *Glycine max*; Ls, *Lactuca sativa*; Mc, *Momordica charantia*; Md, *Malus domestica*; Nt, *Nicotiana tabacum*; Os, *Oryza sativa*; Pa, *Prunus avium*; Pb, *Pyrus x bretschneideri*; Pg, *Punica granatum*; Pp, *Prunus persica*; Pp, *Prunus persica*; Rs, *Raphanus sativus*; Sb, *Sorghum bicolor*; Sei, *Sesamum indicum*; Si, *Setaria italic*; Sl, *Solanum lycopersicum*; So, *Spinacia oleracea*; St, *Solanum tuberosum*; Td, *Triticum dicoccoides*; Vv, *Vitis vinifera*; Zm, *Zea mays*.

# Figure S2

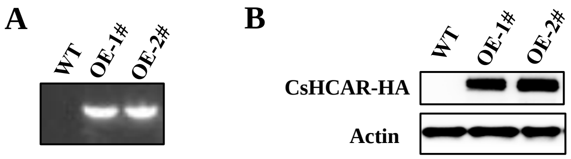


**Figure S2.** Identification of the *CsHCAR* overexpression transgenic tobacco plants. (**A**) Genome PCR identification of the *CsHCAR* overexpression plants. (**B**) Immunoblotting analysis the protein abundance of CsHCAR. WT, wild-type; OE-1# and OE-2#, 2 independent transgenic tobacco overexpression of *CsHCAR*.
