# Supplementary material for: Overexpression of 7-hydroxymethyl Chlorophyll a Reductase from Cucumber in Tobacco Accelerates Dark-Induced Chlorophyll Degradation
Source: Plants (Basel). 2021 Aug 31;10(9):1820. doi: 10.3390/plants10091820 (PMC8465020; doi:10.3390/plants10091820)
Supplement: Supplementary file 1 [file plants-10-01820-s001.zip › Supplementary Materials/Supplemental Tables.pdf]

Table S1. GenBank accession numbers of HCAR used to build phylogenetic tree.

Name of HCAR	GenBank accession numbers
AhHCAR1 (*Arachis hypogaea*)	XP_025633003
AhHCAR2 (*Arachis hypogaea*)	XP_029147852
AtHCAR (*Arabidopsis thaliana*)	NP_171956
BhHCAR (*Benincasa hispida*)	XP_038895859
BoHCAR (*Brassica oleracea*)	XP_013586132
BrHCAR (*Brassica rapa*)	XP_009119272
CisHCAR (*Citrus sinensis*)	XP_024949546
CmHCAR (*Cucumis melo*)	XP_008438432
CpHCAR (*Carica papaya*)	XP_021896897
CsHCAR (*Cucumis sativus*)	XP_004134392
CuMHCAR (*Cucurbita maxima*)	XP_022980288
CumHCAR (*Cucurbita moschata*)	XP_022924576
CupHCAR (*Cucurbita pepo*)	XP_023526945
DcHCAR (*Daucus carota*)	XP_017230458
GhHCAR1 (*Gossypium hirsutum*)	XP_016699579
GhHCAR2 (*Gossypium hirsutum*)	XP_016710081
GmHCAR1 (*Glycine max*)	XP_040869473
GmHCAR2 (*Glycine max*)	XP_003535971
LsHCAR (*Lactuca sativa*)	XP_023767211
McHCAR (*Momordica charantia*)	XP_022157983
MdHCAR (*Malus domestica*)	XP_008360434
NtHCAR1 (*Nicotiana tabacum*)	XP_016491870
NtHCAR2 (*Nicotiana tabacum*)	XP_016496003
OsHCAR (*Oryza sativa*)	XP_015636783
PaHCAR (*Prunus avium*)	XP_021807017
PbHCAR (*Pyrus x bretschneideri*)	XP_009343404
PgHCAR (*Punica granatum*)	XP_031381410
PpHCAR1 (*Prunus persica*)	XP_020411579
PpHCAR2 (*Prunus persica*)	XP_020410804
RsHCAR (*Raphanus sativus*)	XP_018435517
SbHCAR (*Sorghum bicolor*)	XP_002444542
SeiHCAR (*Sesamum indicum*)	XP_020553156
SiHCAR (*Setaria italica*)	XP_004973425
SlHCAR (*Solanum lycopersicum*)	NP_001309778
SoHCAR (*Spinacia oleracea*)	XP_021865457
StHCAR (*Solanum tuberosum*)	XP_006340903
TdHCAR1 (*Triticum dicoccoides*)	XP_037414944
TdHCAR2 (*Triticum dicoccoides*)	XP_037469285
VvHCAR (*Vitis vinifera*)	XP_002285592
ZmHCAR (*Zea mays*)	NP_001131424

Table S2. Primers used for qPCR assays.

Gene name	Forward primer	Reverse primer
NtPPH	5'-GTTATCGTTCCCAGTTTA-3'	5'-AAGTATTGGTGCTGAGTTA-3'
NtPAO	5'-ACCCTAATTCACCTACCC-3'	5'-TTTCATCTATTCTCCCTTC-3'
NtNOL	5'-GGATGGCTTCTTTAGTGC-3'	5'-TGTAAGTAGGCTTTGTTGAT-3'
NtNYC1	5'-TAAACAACGCTGGGACAA -3'	5'-GGATGGCTTCTTTAGTGC-3'
NtRCCR	5'-CTGTGGAGAATCGGCTTGG-3'	5'-ACCTGGGAAGAGGAGTGGC-3'
NtSGR1	5'-TCACTTTGGCTATTTCTC -3	5'-CATCTTCCCTTTAACTTTCT-3
NtRcbL	5'-GTCCCCTGTTGGGATGTACTATT-3'	5'-TGTGAGTTCACGTTCTCATCATC-3'
NtRcbS	5'-TCATTGGATTCGACAACGTG-3'	5'-CACAACCCCTAAAGACAAGACA-3'
NtLhcb1	5'-GCTGCTACAATGGCTCTTT-3'	5'-TGGCGACAGTCTTTCTCA-3'
NtLhcb2	5'-TCCGAGCAAACTCCATCT-3'	5'-CAGTGTCCCATCCGTAAT-3'
NtLhcb4	5'-GAGATGGGCTATGTTGGC-3'	5'-TGGAGAATGGGAGTGGTT-3'
NtActin	5'-CATTGGCGCTGAGAGATTCC-3'	5'-GCAGCTTCCATTCCGATCA-3'
CsHCAR	5'-GTGAAGGCAGATGACGAT-3'	5'-AGTGAATAACGAGCGAAC-3'
CsActin	5'-CAGGAATCCACGAAACTACT -3'	5'-AGACCCTCCAATCCAAACAC-3'

Table S3. Primer sequences for vector construction.

Gene name	Forward primer	Reverse primer
pFGC1008-Cs<br>*HCAR-HA*	5'-ttacaattaccatggggcgcgccATGCA<br>CGCCATTGCCAAC-3'	5'-aacatcgtatgggtaggtaccTTACTTCGT<br>GTTGGAAAGTATGCG-3'
pFGC5941-Cs<br>*HCAR-GFP*	5'-tacaaatctatctctctcgagATGCACG<br>CCATTGCCAAC-3'	5'-ggatccccgggtaccgagctcCTTCGTGTT<br>GGAAAGTATGCG-3'
